# Homoacetogenesis in Deep-Sea *Chloroflexi*, as Inferred by Single-Cell Genomics, Provides a Link to Reductive Dehalogenation in Terrestrial *Dehalococcoidetes*

**DOI:** 10.1128/mBio.02022-17

**Published:** 2017-12-19

**Authors:** Holly L. Sewell, Anne-Kristin Kaster, Alfred M. Spormann

**Affiliations:** a Department of Civil and Environmental Engineering, Stanford University, Stanford, California, USA; b Institute for Biological Interfaces (IBG 5), Karlsruhe Institute of Technology, Karlsruhe, Germany; c Department of Chemical Engineering, Stanford University, Stanford, California, USA; Harvard University

**Keywords:** anaerobic benzoate oxidation, *Anaerolineae*, homoacetogenesis, benzoyl-CoA reductase, *Dehalococcoidia*, Wood-Ljungdahl pathway, reductive dehalogenation

## Abstract

The deep marine subsurface is one of the largest unexplored biospheres on Earth and is widely inhabited by members of the phylum *Chloroflexi*. In this report, we investigated genomes of single cells obtained from deep-sea sediments of the Peruvian Margin, which are enriched in such *Chloroflexi*. 16S rRNA gene sequence analysis placed two of these single-cell-derived genomes (DscP3 and Dsc4) in a clade of subphylum I *Chloroflexi* which were previously recovered from deep-sea sediment in the Okinawa Trough and a third (DscP2-2) as a member of the previously reported DscP2 population from Peruvian Margin site 1230. The presence of genes encoding enzymes of a complete Wood-Ljungdahl pathway, glycolysis/gluconeogenesis, a *Rhodobacter* nitrogen fixation (Rnf) complex, glyosyltransferases, and formate dehydrogenases in the single-cell genomes of DscP3 and Dsc4 and the presence of an NADH-dependent reduced ferredoxin:NADP oxidoreductase (Nfn) and Rnf in the genome of DscP2-2 imply a homoacetogenic lifestyle of these abundant marine *Chloroflexi*. We also report here the first complete pathway for anaerobic benzoate oxidation to acetyl coenzyme A (CoA) in the phylum *Chloroflexi* (DscP3 and Dsc4), including a class I benzoyl-CoA reductase. Of remarkable evolutionary significance, we discovered a gene encoding a formate dehydrogenase (FdnI) with reciprocal closest identity to the formate dehydrogenase-like protein (complex iron-sulfur molybdoenzyme [CISM], DET0187) of terrestrial *Dehalococcoides/Dehalogenimonas* spp. This formate dehydrogenase-like protein has been shown to lack formate dehydrogenase activity in *Dehalococcoides/Dehalogenimonas* spp. and is instead hypothesized to couple HupL hydrogenase to a reductive dehalogenase in the catabolic reductive dehalogenation pathway. This finding of a close functional homologue provides an important missing link for understanding the origin and the metabolic core of terrestrial *Dehalococcoides/Dehalogenimonas* spp. and of reductive dehalogenation, as well as the biology of abundant deep-sea *Chloroflexi*.

## IMPORTANCE

The deep marine subsurface is one of the largest unexplored biospheres on Earth and is widely inhabited by members of the phylum *Chloroflexi*. In this report, we investigated genomes of single cells obtained from deep-sea sediments and provide evidence for a homacetogenic lifestyle of these abundant marine *Chloroflexi*. Moreover, genome signature and key metabolic genes indicate an evolutionary relationship between these deep-sea sediment microbes and terrestrial, reductively dehalogenating *Dehalococcoides*.

## INTRODUCTION

The deep marine subsurface is one of the largest unexplored biospheres on Earth. Recent studies of sediment core samples revealed that it harbors a remarkable abundance and diversity of microbial life forms, most of which are phylogenetically distinct from previously cultured microoorganisms—hence, their phylogenetic affiliation and metabolic characteristics as well as their ecological functions remain largely unknown (1, 2). The activity of subseafloor microorganisms depends mainly on the supply of bioavailable nutrients and energy substrates from the overlying surface (i.e., land and ocean) and/or the underlying lithosphere (i.e., Earth’s crust and mantle). 16S rRNA gene sequence surveys have revealed that the phylum *Chloroflexi* is unusually enriched in deep-sea, diversity-depleted subsurface environments, where in some sediments *Chloroflexi* cell numbers were shown to be nearly equivalent to total bacterial counts (1, 3–5). This general enrichment of *Chloroflexi* raises questions concerning their metabolic niche, the cause for enrichment in marine subsurface sediments, and their role in marine subsurface nutrient cycling.

The *Chloroflexi* phylum contains metabolically diverse microorganisms, including aerobic organotrophs, anoxygenic phototrophs, nitrate reducers, and anaerobic organohalide respirers (6–9). The microorganisms of the latter *Dehalococcoidetes* class-level clade represent an interesting metabolic group as they rely on an obligate dehalogenating lifestyle enabled by a diverse suite of conserved reductive dehalogenases (Rdh) that are of unknown evolutionary origin. Also, the transfer of electrons from hydrogen via a HupL hydrogenase to a reductive dehalogenase is largely unknown and postulated to include a formate dehydrogenase (FDH)-like electron-carrying protein (complex iron-sulfur molybdoenzyme [CISM]) (11, 12). Cultivation-based approaches to a better understanding of the metabolism and the numerical abundance of deep-sea sediment *Chloroflexi* have been hampered by the fact that their natural catabolic substrates are unknown (11). However, single-cell genome analyses have provided insights into the metabolism of these subsurface *Chloroflexi*. Previous work on deep-sea *Dehalococcoides*-related *Chloroflexi* single cells Dsc1 and DscP2 suggested a strictly anaerobic, organotrophic or lithotrophic lifestyle and provided no support for a metabolism based on an obligate activity of catabolic reductive dehalogenases, as found in their terrestrial counterparts (13). The genomes of *Dehalococcoides*-related cells from shallow sediments (DEHC10 and DEHC11, 16.2-m-deep water of Aarhus Bay, 10-cm-deep sediment) appear to indicate an organotrophic lifestyle and potential for ATP synthesis from the conversion of acetyl coenzyme A (CoA) to acetate potentially associated with an incomplete Wood-Ljungdahl pathway (WLP) for homoacetogenesis or by dissimilatory sulfite reduction (14). The genomes of nine *Chloroflexi* single amplified genomes (SAGs) from deep sediments in the Okinawa Trough (seven of the order *Anaerolineales* and one each from the orders *Dehalococcoidales* and *Thermoflexales*) provided genomic evidence through the presence of sugar transporters that those non-*Dehaloccoidales*-related populations may be living heterotrophically (15). Unfortunately, these SAGs were largely incomplete (estimated genome completion from 2% to 32%), which leaves their metabolism largely unidentified.

In this study, we analyzed the genomes of three single-cell-derived genomes taken from deep-sea sediment samples of the Peruvian Margin during the International Ocean Drilling Project (IODP) Leg 201 at sites 1227, 1229, and 1230. Our genome analysis identified two as unclassified members of *Chloroflexi* subphylum I and the third as a member of *Chloroflexi* subphylum II (*Dehalococcoidia*).

## RESULTS

### (1) Sample sites.

We previously analyzed total DNA extracted from deep-sea sediments obtained from the IODP survey (Leg 201) of the Peruvian trench and continental margin for the presence of *Chloroflexi* 16S rRNA and *rdh* genes (16) using PCR and nanoliter-quantitative PCR (qPCR) (13). On the basis of these screening results and the results of previous metagenomic studies of this region (1), we identified samples from continental margin sites 1227 and 1229 and trench site 1230 as promising for future investigation for *Chloroflexi* by single-cell genomics. The surface waters of the Peruvian continental margin are part of an upwelling system that is biologically active and high in total organic carbon, about 2 to 3% by weight (17, 18). Samples were taken from three sites, 1227, 1229, and 1230, at sediment depths 0.3, 1, and 7.3 m below sea floor (mbsf), respectively, corresponding to estimated sediment ages of 1.4, 16, and 64 kiloyears (kyr) (S. D’Hondt, personal communication) (Table 1) (19, 20). All sample sites were located within the sulfate reduction zone. Areal carbon oxidation rates were estimated to be between 2 and 5 µmol cm^−2^ year^−1^ (21, 22). Site 1227 is located in a small, fault-bounded sediment pond within the Trujillo Basin at a water depth of 427 m. Based on maximum alkalinity and dissolved organic carbon (DOC) concentration measurements, site 1227 is considered to be highly biologically active (17). Site 1229 is located on the Peru continental shelf at a water depth of 150.5 m. Previous clone libraries from these sediments revealed that *Chloroflexi* were among the most predominant bacterial groups (5, 23). A more detailed metagenomic study revealed that *Chloroflexi* contributed 12% to 16% to the total gene pool, independent of depth (1). Site 1230 is located at 5,086 m deep on the lower slope of the Peru Trench at the subduction zone between the continental crust and accretionary complex (19).

**TABLE 1:** Sample sites

Site^a^	Hole	Core section	*Chloroflexi* found	Water depth (m)	Sediment depth (mbsf)	Sediment age (kyr)^a^	Acetate concn (μM)^b^	Formate concn (μM)	DOC^c^ (mM)	Sulfate concn (mM)
1227	D	1-1	635N13	427	0.3	16	3	2.25	6.25	30
1229	A	1-1	657A03, 657K04	150	1	1.4	1.25	1.25	21	15
1230	C	115-120	662N06	5,086	7.3	64	1	5	400	24

a Calculated from references 17 and 18.

b Chemical data from interstitial waters at site and depth as reported by reference 98.

c DOC, dissolved organic carbon.

### (2) Single-cell genome recovery, assembly statistics, and phylogeny.

To develop more refined metabolic hypotheses for the lifestyle of deep-sea sediment *Chloroflexi*, we recovered single cells from drilling cores of sites 1227, 1229, and 1230; sorted them into 384-well plates by fluorescence-activated cell sorting; lysed the cells; and amplified the genomes via multiple displacement amplification (MDA) (13, 24). Out of 1,260 wells sorted, three wells containing *Chloroflexi* were identified by PCR screening with broad eubacterial primers targeting the 16S rRNA gene: well 653N14 from site 1227, wells 657A03 and 657K04 from site 1229, and well 662N06 from site 1230. The amplified genomic DNA from these wells was then sequenced. After assembly, we noticed that well 657K04 contained two 16S rRNA genes from two distinct *Chloroflexi* subphyla (25). Each of the 16S rRNA gene sequences was identical to the sequence of either single-cell genome 657A03 or 662N06. Tetranucleotide frequency analyses (TNA) and GC content were sufficient to distinguish the two genomes *in silico* (see Fig. S2 in the supplemental material). Reads mapping to each of these two genomes in well 657K04 were coassembled with the reads from corresponding well 657A03 or 662N06, depending on 16S rRNA gene sequence identity and TNA. The combined assembly of 657A03 and 657K04 is referred to as DscP3 (deep-sea single cell, population 3) (Table S1). Since the 16S rRNA gene sequence of 662N06 is identical to DscP2 (13), the combined assembly of 662N06 and 657K04 is referred to as DscP2-2. As the genomic content from well 653N14 was from a single cell, the assembly of the genomic data was named Dsc4 (deep-sea single cell 4).

10.1128/mBio.02022-17.2 FIG S1  Sample site locations. Download FIG S1, DOCX file, 0.4 MB. Copyright © 2017 Sewell et al. 2017 Sewell et al. This content is distributed under the terms of the Creative Commons Attribution 4.0 International license.

10.1128/mBio.02022-17.3 FIG S2  Contamination screen. Download FIG S2, DOCX file, 0.3 MB. Copyright © 2017 Sewell et al. 2017 Sewell et al. This content is distributed under the terms of the Creative Commons Attribution 4.0 International license.

10.1128/mBio.02022-17.10 TABLE S1  Assembly statistics. Download TABLE S1, DOCX file, 0.1 MB. Copyright © 2017 Sewell et al. 2017 Sewell et al. This content is distributed under the terms of the Creative Commons Attribution 4.0 International license.

**FIG 1:**
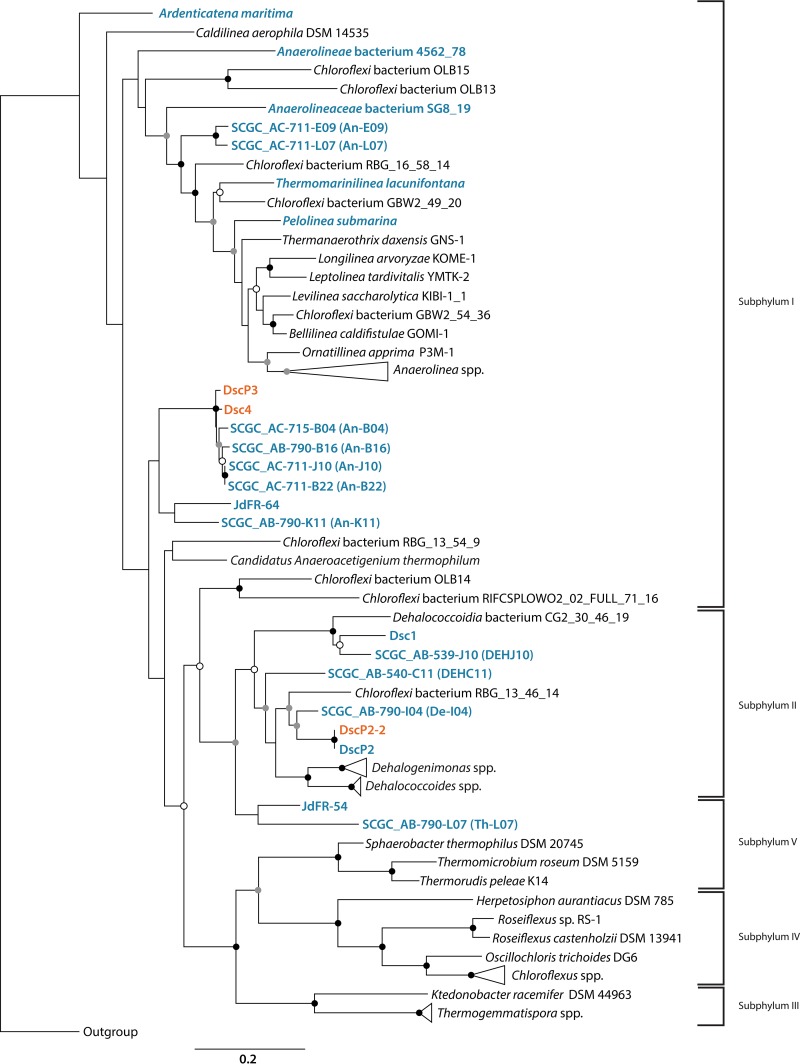
Rooted maximum likelihood tree of 16S rRNA gene. Evolutionary distance tree of the bacterial phylum *Chloroflexi* derived from comparative analysis of 16S rRNA gene sequences showing the phylogenetic relationship between members of the phylum with *Escherichia coli* as the outgroup. The sequences were aligned with the method described in reference 89 and masked using Gblocks (96), and the phylogenetic tree was constructed using the maximum likelihood method using PhyML (97) and the TN93 substitution model. Nodes highly supported by bootstrap resampling (100 replicates) are represented by black (≥90%), gray (≥70%), or white (≥50%) circles, respectively. Names in orange are genomes presented in this study, and those in blue are other marine *Chloroflexi*.

Classification of the 16S rRNA gene sequences revealed that the single-cell-derived genomes belong to cells of *Chloroflexi* subphyla I and II (Fig. 1) (26). DscP3 and Dsc4 are unclassified *Chloroflexi* of subphylum I, most closely related to single cells An-B04, An-B16, An-B22, and An-J10 from the Okinawa Trough (15). DscP2-2 is a member of the class-level clade *Dehalococcoidetes* of *Chloroflexi* subphylum II with a 16S rRNA gene sequence identical to the previously reported DscP2; these populations are most closely related to Okinawa Trough single cell De-I04 and terrestrial RBG_16-57-8, RBG_13_46_14, and RBG_16_51_9 obtained from Rifle, CO, aquifer sediments (13, 15, 27) (Fig. 1).

Genome completeness estimates using tRNAs and marker genes from CheckM (see Table S1, reference 28) predicted the size of the genomes of the subphylum I members DscP3 and Dsc4 to be between 2 and 2.7 Mb (conservative completeness estimates of 64% and 49%, respectively), significantly smaller than the 3.2- to 3.5-Mb genome size estimated for closely related An-B04, An-B16, An-B22, and An-J10 (15), though the discrepancy is likely due to the incompleteness of these genomes. On the other hand, for DscP2-2 we estimate the genome size to be around 1 to 1.3 Mb (36% completeness), similar to the 1.38-Mb genome size previously predicted for population DscP2 (13). Below, we report our metabolic reconstruction from single-cell genomic data for DscP3 and Dsc4 first, followed by DscP2-2.

### (3) Predicted Metabolism of DscP3 and Dsc4.

We analyzed the genomes of DscP3 and Dsc4 with respect to catabolic pathways. Central metabolic pathways are predicted to include complete pathways for glycolysis/gluconeogenesis and the Wood-Ljungdahl pathway (WLP, see below). The annotation of multiple sugar transporters suggests external carbohydrates as substrates for the glycolytic pathway, and possibly the consumption of the released reducing equivalents by the WLP. The presence of Rnf (rhodobacter nitrogen fixation) and Hdr-IFO (heterodisulfide-reductase-associated ion-translocating Fd:NADH oxidoreductase) provides potential means of chemiosmotic energy conservation. No evidence for respiration of inorganic electron acceptors was found. Because of the presence of these genes, we hypothesized that DscP3 and Dsc4 have the genomic potential for a homoacetogenic lifestyle.

### (3.1) (i) Wood-Ljungdahl pathway.

Homoacetogenic bacteria are anaerobic, facultative autotrophs that are able to grow by oxidation of organic or inorganic substrates coupled to the reduction of carbon dioxide to produce acetate via the Wood-Ljungdahl pathway (WLP). Although most known homoacetogenic bacteria are found within the genera *Clostridium* and *Acetobacterium*, this metabolism is present in 23 different bacterial genera (29). Despite their phylogenetic diversity, the hallmark of a homoacetogenic metabolism is the use of the WLP for CO_2_ reduction to acetate coupled to a membrane-bound enzyme complex for energy conservation (29–31). The ubiquity of the homoacetogenic bacteria in diverse environments can be attributed to the metabolic flexibility of these microorganisms. Electron donors for the WLP include a large variety of organic and inorganic substrates including formate, hexoses, pentoses, cellulose, alcohols, hydrogen (H_2_), and CO (30). Homoacetate fermentation, more commonly referred to as homoacetogenesis, is a form of acetogenic metabolism that uses the Embden-Meyerhof-Parnas (glycolytic) pathway for the oxidation of glucose to 2 molecules of acetate and 2 molecules of CO_2_ which are subsequently reduced to an additional molecule of acetate (31).

The WLP consists of two branches—the methyl-branch and the carbonyl-branch. In the forward (reductive) direction the methyl group of acetate is formed in the methyl-branch through the three-step reduction of CO_2_ and the carbonyl-branch generates the carbonyl group of acetate via the one-step reduction of a second molecule of CO_2_. In the methyl-branch, one molecule of CO_2_ is reduced to formate, catalyzed by a formate dehydrogenase. Formyl-tetrahydrofolate synthetase (Fhs) covalently binds the formyl group to tetrahydrofolate (THF) coupled to hydrolysis of ATP to produce formyl-THF. The bifunctional enzyme, methenyl-THF cyclohydrolase/methylene-THF dehydrogenase (FolD) abstracts a water molecule from formyl-THF to yield methenyl-THF, which is then reduced to methylene-THF. The final reduction step is catalyzed by methylene-THF reductase (MetF), which produces methyl-THF. The methyl group is then transferred by a methyltransferase and a corrinoid iron-sulfur protein to CO dehydrogenase/acetyl-CoA synthase (CODH/ACS) which also catalyzes the one-step reduction of CO_2_ to CO in the carbonyl-branch. The CODH/ACS catalyzes the final carbonylation of methyl-THF by CO to form acetyl-CoA.

The WLP is utilized by a wide variety of anaerobic microorganisms for both C1 metabolism and energy conservation by coupling folate-mediated C1 metabolism to either CO_2_ reduction (forward) or acetate oxidation (reverse). In fact, the WLP is considered to be one of the most ancient pathways for biomass and ATP production (30, 31, 100). In the forward direction, homoacetogenic bacteria utilize the WLP for acetate production and energy conservation. In some sulfate-reducing bacteria, the WLP operates in reverse by coupling the exergonic reduction of sulfate to sulfide to the oxidation of acetate (35). The incomplete WLP found in *Dehalococcoides* spp. has been implicated in the unconventional strategy of generating methyl-tetrahydrofolate for methionine biosynthesis (101).

As we found in the genomes of DscP3 and Dsc4 genes encoding a complete WLP, we compared in more detail the genes encoding the predicted pathway enzymes and associated operon architectures in *Chloroflexi* DscP3 and closely-related Dsc4 with those of two well-studied homoacetogenic bacteria, the Gram-positive *Moorella thermoacetica* and *Acetobacterium woodii* (31, 32). While both of these homoacetogenic bacteria carry the genes of the catabolic WLP for acetate formation from two CO_2_ with H_2_ as electron donor, they differ in key genes and mechanisms for energy conservation (31).

Energy conservation in *M. thermoacetica* is based on H_2_-cycling and a H_2_-dependent NADP^+^ reduction, where both H_2_-cycling and NADPH oxidation are associated with CO_2_ reduction in the WLP. In the net energy-conserving module of *M. thermoacetica*, NADPH is bifurcated into reduced ferredoxin and NADH; the former being used for CO_2_ reduction to CO. NADH is further bifurcated by the hexaheteromeric HdrABC-MvhD-MetVF complex (Moth_1191-1196) to reduce methylene-THF and an unknown, low redox potential electron carrier, which is speculated to reduce H^+^ to H_2_ via a membrane-associated, H^+^-pumping hydrogenase (Ech) (103). Thus, a HdrABC-MvhD-MetVF methylene-THF reductase, a NADPH-bifurcating Nfn, and an Ech are the defining enzymes for an idiosyncratic catabolism of *M. thermoacetica*.

In contrast, *Acetobacterium woodii* contains a linear, H_2_-dependent energy metabolism, where a soluble tetrameric electron-bifurcating hydrogenase (HydABCD) catalyzes the oxidation of hydrogen to the generation of NADH and a reduced ferredoxin (31). Energy conservation is mediated by a Na^+^-translocating Rnf complex, coupling the oxidation of reduced ferredoxin to the reduction of NAD^+^. The reducing equivalents of NADH are subsequently funneled into the methyl-branch of the WLP. Homoacetogenic metabolism from glucose in A. woodii involves the production of 2 acetate, 2 NADH, and 2 reduced ferredoxin. Both NADH and 1 reduced ferredoxin are utilized in the WLP. The Rnf complex transfers 0.5 ferredoxin to generate 0.5 NADH, which, combined with the remaining 0.5 ferredoxin, generates hydrogen via HydABCD in the electron-confurcating direction (102). Thus, key enzymes of an *A. woodii* homoacetogenic catabolism are an NADH-dependent methylene-THF reductase, an electron-bifurcating hydrogenease, an Rnf, and the absence of Nfn (30, 37, 106, 107).

**FIG 2:**
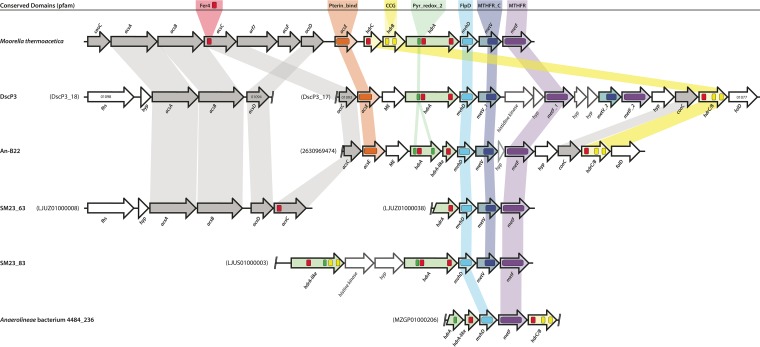
Gene order and domain composition between the methylene tetrahydrofolate reductase and associated genes from *Moorella thermoacetica* and marine *Chloroflexi*. Several sequenced *Anaerolineae* populations and single cells from marine sediments, including DscP3, share similarities in operon structure with *Moorella thermoacetica*. An-B22 is a single-cell genome from the Okinawa Trough (15), SM23_63 and SM23_83 are from White Oak River metagenomes (33), and *Anaerolineae* bacterium 4484-236 is from a Guayamas Basin metagenome (NCBI accession no. MZGP00000000). Dashed double lines indicate the end of a contig. Unlabeled genes are nonconserved hypothetical proteins. Genes colored gray are upstream of the *hdrABC-metVF-mvhD* operon in *M. thermoacetica*. Fer4 (red) is the 4Fe-4S-binding domain (PF00037); Pterin_bind (orange) is the pterin-binding protein family (PF00809); CCG (yellow) is the cysteine-rich domain (PF02754); Pyr_redox_2 (green) is the pyridine nucleotide-disulfide oxidoreductase family, which contains a small NAD(P)H-binding domain within a larger FAD domain (PF07992); FlpD (blue) is the methyl-viologen reducing hydrogenase delta subunit family containing 4 conserved cysteine residues (PF02662); MTHFR_C (indigo) family is the C-terminal domain of MetF containing the FAD-binding site (PF12225); MTHFR (violet) is the MetF family, including the FAD-binding domain and TIM barrel (PF02219).

The DscP3 genome contains a *hdrABC-mvhD-metVF* gene cluster in an operon similar to that of *M. thermoacetica* (Fig. 2) (103). Unlike *M. thermoacetica* however, the *hdrC* gene was present in a translational fusion with *hdrB* as found also in some sulfate-reducing bacteria (104) and methanogens without cytochromes such as *Methanothermobacter marburgensis* (36, 105). A BLAST comparison of this DscP3 operon to genomes of other sequenced *Chloroflexi* and *Chloroflexi*-containing metagenomes found similar gene clusters in contigs from shotgun metagenomes of the White Oak River Basin (33) as well as the SAG An-B22, from the Okinawa Trough (15) indicating conservation of the herein-identified *hdrABD-mvhD-metVF* gene cluster among marine *Chloroflexi*. Uniquely, DscP3 has two non-identical sets of *metV* and *metF* genes sharing only 42.8% (bit score, 257) and 40.9% (bit score, 71.6) amino acid sequence similarity, respectively. The first methylene-THF reductase, MetF_1, has 93% amino acid similarity to the MetF of An-B22 and, in a comparison to *M. thermoacetica* and *A. woodii*, has an amino acid sequence identity closer to *M. thermoacetica* (54.5%; bit score, 367) than to *A. woodii* (38.3%; bit score, 236). The second copy, MetF_2, is closest in sequence identity to the MetF of *A. woodii* (56.8%; bit score, 329) relative to *M. thermoacetica* (42.23%; bit score, 244). In the genomes of homoacetogens, *metV* and *metF* are arranged in sequence; however, genes encoding a putative two component regulatory system, *phoBR*, flank *metF_1* in DscP3. No other known *Chloroflexi* clade contains the *metV* gene required for MetF function in homoacetogens, making the *Moorella*-type HdrABC-MvhD-MetVF complex of DscP3 unique to marine members of *Chloroflexi* subphylum I.

The bifunctional methenyl-THF cyclohydrolase/methylene-THF dehydrogenase (*folD*) of DscP3 has 58% (bit score, 329) amino acid identity to that gene of *M. thermoacetica* (Moth_1516) and 54% (bit score, 252) identity to the *folD* of *Methanosarcina barkeri* (Mbar_82315). Experimental evidence showed that FolD in these two microorganisms is specific for NADPH as electron donor rather than NADH as in *A. woodii* (AWO_RS04705) or *Clostridium ljungdahlii* (AWO_RS04705) (31, 38, 39, 108). Because of the higher sequence similarity between NADPH-specific FolD of *M. barkeri* and *M. thermoacetica* to those of DscP3 and Dsc4, we hypothesize that *folD* in the herein-identified deep-sea *Chloroflexi* are likewise specific for NADPH.

### (3.2) (ii) Proton-translocating membrane-associated complexes in DscP3 and Dsc4.

The genomes of the subphylum I *Chloroflexi* DscP3 and Dsc4 contain genes encoding an Rnf complex, a heterodisulfide-reductase-associated ion-translocating Fd:NADH oxidoreductase (Hdr-IFO), a partial NADH-ubiquinone oxidoreductase (Nuo), and an F-type ATP synthase.

### (3.2.1) *Rhodobacter* nitrogen fixation complex.

The *Rhodobacter* nitrogen fixation (Rnf) complex couples the exergonic reduction of NAD^+^ with reduced ferredoxin (Fd) to proton or sodium translocation and can function anabolically in the energy-consuming, Fd-reducing direction, such as in *Rhodobacter capsulatus*, *Pseudomonas*, *Azotobacter*, and *Escherichia* species, or catabolically in the energy-conserving, NAD^+^-reducing direction, as in *Acetobacterium woodii*, *Clostridium kluyveri*, and *Methanosarcina acetivorans* (40–42). The flavoprotein maturation enzyme, ApbE, is frequently found directly adjacent to the *rnf* operon, and in *Rhodobacteria* and sulfate reducers, it is often annotated as *rnfF* (43, 44). Based on operon organization, Rnf can be classified into three distinct types (Fig. S5) (42). The *rnfABCDGE* type is found in *Rhodobacter*, *Pseudomonas*, *Azotobacter*, or *Escherichia* and seems to be primarily of anabolic function to generate reduced Fd from NADH for biosyntheses (109, 110, 111). The *rnf*(*B*)*CDGEA* organization is found in species of *Chlorobium*, *Bacteroides*, and *Prosthecochloris* (42). The third configuration, *rnfCDGEAB*, is found in *Acetobacterium woodii*, *Methanosarcina acetivorans*, members of the *Clostridia* genera, and some *Desulfovibrio* species, among others (48, 107, 112, 113, 114). In *Clostridium kluyveri*, *M. acetivorans*, and *A. woodii*, Rnf (*rnfCDGEAB*) is the key complex in the energy conservation pathway during homoacetogenesis (31). DscP3 and Dsc4 both harbor genes for a complete RnfCDGEAB directly preceded by ApbE (Fig. S5).

### (3.2.2) Heterodisulfide-reductase-associated ion-translocating Fd:NADH oxidoreductase.

In DscP3 and Dsc4 (as well as in An-B16), genes are present encoding a putative heterodisulfide-reductase-associated ion-translocating Fd:NADH oxidoreductase (Hdr-IFO) postulated to be involved in reverse electron transport in *Syntrophobacter fumaroxidans* (45). This gene cassette encodes HdrABC subunits and two putative oxidoreductase domain-containing proteins indicative of Fd:NAD(H) oxidoreductase. The genes encoding Hdr-IFO are conserved among bacteria capable of syntrophic metabolism: *M. thermoacetica* DSM 512 (Mothe_RS03705 to -RS03675), *Clostridium halophilum* (SAMN02194393_03034 to -03037), *Desulfovirgula thermocuniculi* (G4G4_RS0108870 to -RS0108895), *Thermincola potens* (TherJR_1949 to -1955), *Syntrophoaceticus schinkii* strain Sp3 (SSCH_160001 to -160008), *Syntrophomonas wolfei* (SWOL_RS02005 to -RS02050), *Syntrophorhabdus aromaticivorans* strain UI (SynarDRAFT_0715 to -0709), and *Desulfovibrio vulgaris* (DVU2404 to -2399) (Fig. S6). In a study of *Desulfovibrio vulgaris* strain Hildenborough, it was revealed that this gene cassette, but not the Rnf operon, was expressed when grown in a lactate-degrading syntrophic coculture with a hydrogenotrophic methanogen (46–49). A previously published survey of publicly available genomes of bacteria capable of syntrophic metabolism indicated that, with the exception of *Desulfovibrio*, the genomes of syntrophic bacteria contain either Rnf or Hdr-IFO (45). In syntrophic bacteria, Rnf and Hdr-IFO are thought to be involved in reverse electron transport, using NADH and an ion motive force to reduce Fd (45).

### (3.2.3) NADH-ubiquinone oxidoreductase-like complex.

A functional NADH-ubiquinone oxidoreductase (Nuo) operon generally contains 14 subunits in three modules, NADH-binding (N), proton-translocating (P), and quinone-reducing (Q) modules, which are hypothesized to have emerged through the combination of separate functional modules (50, 51). Like the genome of DEHC11 from Aarhus Bay, the genomes of DscP3 and Dsc4 lack the genes for the Nuo N module (*nuoE* to -*G*), leaving only 11 subunits in the operon (52). The 11-subunit Nuo complex bears a resemblance to Ech, except that it lacks the [NiFe]-binding motifs in subunits *nuoB* and *nuoD* that are required for hydrogenase function (51). The N module, however, is frequently absent in *Eubacteria* and *Archaea*, and the 11-subunit Nuo may couple with another complex encoded elsewhere in the genomes of these microorganisms (51). The partial genome most closely related to DscP3 and Dsc4, An-B22, does contain both an 11-subunit Nuo and the *nuoEFG* genes for a complete NADH module in a separate operon, indicating that a Nuo complex may be part of the core metabolism of deep-sea *Chloroflexi*.

### (3.3) (iii) Genomic signatures for electron donors for the Wood-Ljungdahl pathway.

In homoacetogenic metabolism, reducing equivalents for the WLP are generated by oxidizing glucose to two molecules of acetate and two molecules of CO_2_ via glycolysis, pyruvate:ferredoxin oxidoreductase (PFOR), phosphotransacetylase, and acetate kinase (31). In our single-cell assemblies, DscP3 and Dsc4, we found all required genes for glycolysis and gluconeogenesis except those for carbohydrate activation. The presence of PFOR, phosphotransacetylase, and acetate kinase provides a link for acetate formation from pyruvate. Putative α-glucoside ABC transporters identified as carbohydrate-binding module family 50, chitin or peptidoglycan specific, were identified in the genomes of both DscP3 and Dsc4, indicating a possible input for the glycolytic pathway. Genomes of closely related *Chloroflexi*, An-B10 and An-B22, contain genes for xylose, hexose, and multiple-sugar ABC transporters (15).

The genome of DscP3 contains an l-lactate dehydrogenase (LDH), and both DscP3 and Dsc4 contain l-lactate permease. The pathway for acetogenic fermentation of lactate has recently been described in *A. woodii* (115). In *A. woodii*, the LDH forms a complex with EtfAB and uses flavin-based electron confurcation to drive the highly endergonic NAD^+^-dependent oxidation of lactate with the exergonic reduction of NAD^+^ from reduced ferredoxin. The oxidation of pyruvate proceeds as in heterotrophic fermentation, yielding 1 acetate, 1 CO_2_, and 1 ATP molecule per pyruvate. The stoichiometry of acetogenic fermentation of lactate by *A. woodii* is the production of 2 acetate, 2 CO_2_, 2 ATP, and 4 NADH molecules per molecule of lactate (115). The WLP serves to balance the reducing equivalents produced. Unlike heterotrophic fermentation, this involves the input of energy to generate reduced ferredoxin and H_2_ from 2 NADH. The electron-bifurcating hydrogenase can produce H_2_ from 0.5 molecule of reduced ferredoxin and 0.5 molecule of NADH. The remaining 1.5 molecules of NADH are used by Rnf to produce the necessary 1.5 molecules of reduced ferredoxin coupled with the transport of a proton or sodium ion with the chemiosmotic gradient. As mentioned above, DscP3 and Dsc4 contain lactate dehydrogenase, EtfAB, and Rnf. To provide the reducing power for acetogenic lactate fermentation, an electron-bifurcating hydrogenase or formate dehydrogenase (described below) is needed.

In order to identify other potential electron donors for the WLP, we examined the genomes of DscP3, Dsc4, and related marine *Chloroflexi* for the presence of hydrogenases and formate dehydrogenases. Although the genomes of DscP3 and Dsc4 are incomplete, we found no genomic evidence for the presence of hydrogenases or hydrogenase expression/maturation genes. Other closely related, yet also incomplete, sequenced *Chloroflexi* single-cell genomes, An-B16, An-J10, and An-L06, do contain hydrogenase expression/maturation genes *hypABCDEF* while An-B22 does not (15). This makes it difficult to determine whether hydrogenase genes in DscP3 and Dsc4 are either absent or just not amplified as a result of the bias of the MDA reaction. However, both the DscP3 and Dsc4 genomes do encode multiple putative formate dehydrogenases.

### (3.3.1) Formate dehydrogenases.

Three distinct types of formate dehydrogenases (FDHs) were found to be encoded by the genomes of DscP3 and Dsc4: an NAD(P)-dependent FDH (NAD-FdhAB), a formate:quinol oxidoreductase (FdnGHI), and a heterodisulfide reductase-associated FDH (HdrBCA-MvhD-FdhBA).

### (3.3.1.1) NAD-dependent formate dehydrogenase.

A putative heterodimeric NAD(P)-dependent FDH (FdhAB) complex was identified in the genome of DscP3. Previously, the genome of SAG An-B22 was also hypothesized to contain genes for an NAD-dependent FDH (15). The α subunits of the NAD-FdhAB from DscP3 and An-B22 share 78% amino acid identity, but the sequence of An-B22 contains a selenocysteine residue in the active site (15). The cytoplasmic, selenocysteine-containing, NADP-dependent FdhAB of *M. thermoacetica* (MOTH_2312 to -2314) is involved in the generation of formate in the first step of the CO_2_ reduction of the methyl branch of the Wood-Ljungdahl pathway (10). The α subunits of FdhAB in DscP3 and the NADP-dependent FdhAB of *M. thermoacetica*, respectively, share 43% (bit score, 961) amino acid identity; however, the β subunits are dissimilar. FdhB of *M. thermoacetica* contains a 4Fe-4S cluster-binding domain, a ferredoxin-binding domain, and an NADP-binding domain. On the other hand, the β subunit of FdhAB from DscP3 contains a thioredoxin-like 2Fe-2S ferredoxin, an NAD(P)-binding domain, an flavin mononucleotide (FMN)-binding domain, a soluble ligand-binding domain, and a 4Fe-4S cluster-binding domain. It is unclear whether or not the predicted NAD(P)-binding site is specific to NAD^+^ or NADP^+^. The best BLASTP hit for the β subunit of the FdhAB from DscP3 is to the NADH-ubiquinone oxidoreductase subunit F (NuoF) of *Anaerolinea thermophila* UN-1 with 53% (bit score, 519) amino acid identity.

**FIG 3:**
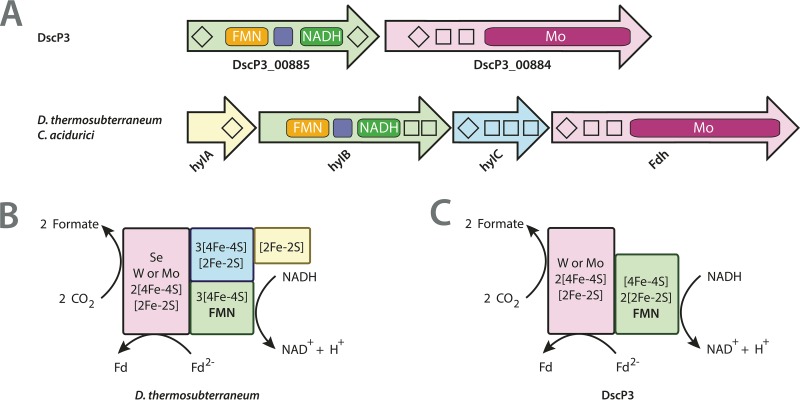
NAD-dependent formate dehydrogenase. (A) Subunit similarity between the NAD-dependent formate dehydrogenase from DscP3 and the electron-bifurcating/confurcating formate dehydrogenases of *Clostridium acidurici* and *Desulfotomaculum thermosubterraneum* (54, 55). (B) Predicted electron-bifurcating formate dehydrogenase from *D. thermosubterraneum*. (C) Predicted NAD-dependent formate dehydrogenase from single-cell population DscP3.

The best BLASTP hit for the α subunit was to the FdhA of the thermophilic sulfate-reducing bacterium *Desulfotomaculum thermosubterraneum* (60%; bit score, 1057). The NAD-dependent FDH of *D. thermosubterraneum* (BUA65_RS16050 to -RS16065) and other *Desulfotomaculum* species is hypothesized to be electron confurcating, driving the unfavorable oxidation of NADH generated during propionate degradation to the exergonic oxidation of ferredoxin to produce formate (54). A similar NAD-dependent FDH complex (HylCBA-FdhA) in *Clostridium acidurici* (Curi_c29380 to -c29410) functions in the reverse (bifurcating) direction during uric acid fermentation (55). The bifurcating/confurcating HylCBA-FdhA complex in *C. acidurici* (Curi_c29380 to -c29410) and that in *D. thermosubterraneum* (BUA65_RS16050 to -RS16065) each consist of four subunits: HylC, HylB, HylA, and the catalytic Fdh α subunit, FdhA. HylC contains a 2Fe-2S cluster-binding domain; HylB contains a 2Fe-2S iron-sulfur-binding domain, an NAD-binding domain, a soluble ligand-binding domain, an FMN-binding domain, and two 4Fe-4S cluster-binding domains; and HylA contains one 2Fe-2S and three 4Fe-4S cluster-binding domains. FdhA contains one 2Fe-2S and one 4Fe-4S cluster-binding domain, a selenocysteine, and a molybdopterin-binding domain. The genome of DscP3 contains genes for the two subunits FdhA and FdhB, which bear resemblance to HylA and the NAD-binding subunit, HylB, respectively, with a thioredoxin-like 2Fe-2S cluster-binding domain instead of two 4Fe-4S cluster-binding domains (Fig. 3). The similarities between the subunits of the heterotetrameric electron-bifurcating/confurcating HylCBA-FdhA from *C. acidurici* and *Desulfotomaculum* spp. indicate that the predicted heterodimeric NAD-dependent FDH of DscP3 might also be electron bifurcating/confurcating. If FdhAB is an electron bifurcating Fdh, we hypothesize that formate could be oxidized to CO_2_ during acetate oxidation via the WLP, and as an electron confurcating Fdh, CO_2_ could be reduced to formate as the first intermediate of the methyl branch of the WLP. This bifurcating/conforcating formate dehydrogenase may also provide a link between lactate fermentation and the WLP, similar to the electron-bifurcating hydrogenase in *A. woodii* (115).

### (3.3.1.2) Formate:quinol oxidoreductase.

The formate:quinol oxidoreductase complex, often referred to as formate dehydrogenase-N (FdnGHI), is a three-subunit, membrane-integral protein found in the genomes of both DscP3 and Dsc4. FdnI is the catalytic subunit, encompassing a putative molybdopterin-binding domain containing a TAT-signal peptide sequence; FdnH contains two 4Fe-4S cluster domains; and FdnG is predicted to be membrane bound and to contain two cytochrome *b* domains (56). The FdnI subunit of DscP3 is an incomplete sequence, being cut off by the end of a contig, but Dsc4 contains a second FdnI subunit directly downstream of FdnGHI, sharing 48% amino acid similarity with the first. The first FdnI sequence, FdnI_1, displays highest similarity to the α subunit of the membrane-bound FDH-like oxidoreductase complex in *Dehalogenimonas lykanthroporepellens* (Dehly_0443) and *Dehalococcoides ethenogenes* 195 (DET0187) at 56% (bit score, 1,104) and 49% (bit score, 752) amino acid identity, respectively (Fig. S3). The second FdnI, FdnI_2, is predicted to be selenocysteine containing and has 68% (bit score, 1,067) amino acid similarity to *Bacillus* sp. strain NC2-31.

10.1128/mBio.02022-17.4 FIG S3  Sequence alignment of formate dehydrogenase-like enzymes. Download FIG S3, DOCX file, 0.2 MB. Copyright © 2017 Sewell et al. 2017 Sewell et al. This content is distributed under the terms of the Creative Commons Attribution 4.0 International license.

The FdnH and FdnG subunits of the FdnGHI complex in Dsc4 are most similar to the iron-sulfur subunit of the polysulfide reductase from *Calderihabitans maritimus* (KKC1_02500) (58%; bit score, 320) and the cytochrome *b*-containing integral membrane subunit (SAMN06269301_1443) of an FDH from *Geobacter* sp. strain DSM 9736 (41%; bit score, 248), respectively (57). The FdnH and FdnG subunits from DscP3 are most similar to those in *Bacillus massilosenegalensis* with 46% (bit score, 160) amino acid identity for the iron-sulfur β subunit and 39% (bit score, 244) for the cytochrome *b* subunit. In *Bacillus*, FdnGHI is hypothesized to mediate the effects of acidity through the oxidation of formate, generating NAD(P)H which transfers electrons to the electron transport chain through cytochrome *b* (58). Two membrane-associated FDHs in *Escherichia coli*, FdnGHI and FdoGHI, were found to exhibit substrate promiscuity and to have hydrogen-oxidizing activity (56). Similar to the membrane-associated FDH in *Bacillus* spp. and *E. coli*, the FdnGHI of DscP3 and Dsc4 may likely be a formate- and/or hydrogen-oxidizing enzyme.

Interestingly, in terrestrial *Dehalococcoides* spp. the operon encoding the FDH-like complex consists of two genes: one encoding a formate dehydrogenase catalytic subunit homologue with similarities to FdnI and the second encoding a membrane-bound subunit homologous to FdnH but lacking the cytochrome *b* domains. The operon is missing an iron-sulfur cluster-containing FdnH homologue and is instead hypothesized to associate with the Hup hydrogenase complex (DET0110 to -0112) as a part of quinone-independent organohalogen respiration via reductive dehalogenases (11, 12, 53, 59). The FdnI homologue of *Dehalococcoides* spp. contains a serine residue instead of a cysteine or selenocysteine at a key position in the active site, which is hypothesized to be the cause for the lack of formate dehydrogenase activity (53). Notably, the FDH-like protein of *Dehalococcoidetes* and the associated 4Fe-4S cluster-containing subunit of the Hup operon are closest in sequence similarity to the FdnI (48%; bit score, 262) and FdnH (35%; bit score, 191) subunits, respectively, from Dsc4. The FDH-like complexes of *Dehalogenimonas* and *Dehalococcoides*, while membrane bound, have no cytochrome *b* subunit. *Dehalogenimonas*, *Dehalococcoides*, and other subphylum II *Chloroflexi* do not synthesize quinones. Menaquinones, however, have been recovered from cell extracts of facultative anaerobic members of *Chloroflexi* subphylum I, *Ardenticatenia* and *Caldilinea* (60, 61). Like these other members of subphylum I, we hypothesize that DscP3 and Dsc4 are capable of quinone-dependent electron transfer as supported by the presence of the genes *menA* and *ubiE/menG*, which catalyze the synthesis of menaquinone from 1,4-dihydroxy-2-naphthoate.

### (3.3.1.3) Heterodisulfide reductase-associated formate dehydrogenase.

A selenocysteine-containing FDH was found downstream of the *mvhD*, *hdrA*, and *hdrBC* genes that encode a potential HdrBCA-MvhD-FdhBA similar to that of *Desulfobacula toluolica* Tol2 (TOL2_RS06275 to -RS06300), an aromatic-compound-degrading, sulfate-reducing bacterium from marine sediment (Fig. S4) (62). It has been observed in methanogenic metabolism that FDH can couple with complexes containing HdrA-like proteins for electron bifurcation (63). FdhA of this putative complex is selenocysteine containing and is, with 59% (bit score, 852) amino acid identity, most closely related to the α subunit of the four-subunit NAD-dependent FDH of the halophilic anaerobe *Selenihalanaerobacter shriftii* (B5D41_RS09265). The β subunit, FdhB, contains an iron-sulfur cluster-binding domain and has highest amino acid similarity at 40% (bit score, 206) to the FdhB of *D. toluolica* Tol2. Interestingly, the reductive dehalogenase RdhA sequence found in DEHC11 from Aarhus Bay was found to be most similar to a homologue found in *D. toluolica* Tol2 (14).

10.1128/mBio.02022-17.5 FIG S4  Heterodisulfide reductase-associated formate dehydrogenase. Download FIG S4, DOCX file, 0.1 MB. Copyright © 2017 Sewell et al. 2017 Sewell et al. This content is distributed under the terms of the Creative Commons Attribution 4.0 International license.

### (3.4) Anaerobic benzoate oxidation.

**FIG 4:**
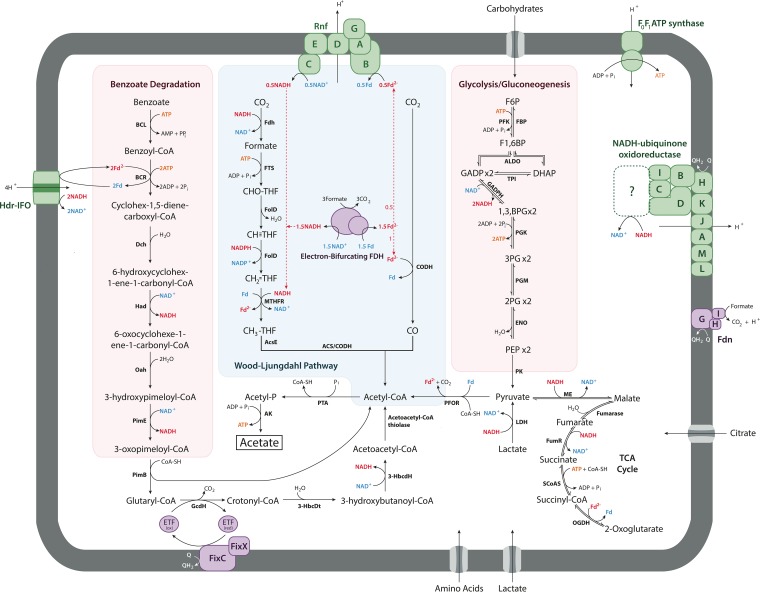
Reconstructed energy metabolism of DscP3 and Dsc4. All pathways found to be supported by genomic evidence in DscP3 and/or Dsc4. Reduced electron carriers are shown in red, oxidized electron carriers in blue, and ATP in orange. Abbreviations: F6P, fructose-6-phosphate; PFK, phosphofructokinase; FBP, fructose-1,6-bisphosphatase; F1,6BP, fructose bisphosphate aldolase; TPI, triose phosphate isomerase; PGK, phosphoglycerate kinase; 3PG, 3-phosphoglycerate; 2PG, 2-phosphoglycerate; PGM, phosphoglycerate mutase; THF, tetrahydrofolate; Fd, ferredoxin; ME, malic enzyme; GADP, glyceraldehyde-3-phosphate; GADPH, glyceraldehyde-3-phosphate dehydrogenase; DHAP, dihydroxyacetone phosphate; PEP, phosphoenolpyruvate; PK, pyruvate kinase; LDH, lactate dehydrogenase; FumR, fumarate reducatse; SCoAS, succinyl-CoA synthase; OGDH, 2-oxoglutarate dehydrogenase; Fdn, formate dehydrogenase-N; PFOR, pyruvate-ferredoxin oxidoreductase; PTA, phosphotransacetylase; AK, acetate kinase; Rnf, *Rhodobacter* nitrogen fixation complex; FdhAB, formate dehydrogenase H; FTS, 10-formyltetrahydrofolate synthetase; FolD, bifunctional 5,10-methylene-tetrahydrofolate dehydrogenase/5,10-methylene-tetrahydrofolate cyclohydrolase; MTHFR, 5,10-methylenetetrahydrofolate reductase; AcsE, methyltetrahydrofolate:corrinoid/iron-sulfur protein methyltransferase; CoFeSP, corrinoid iron-sulfur protein; ACS, acetyl-CoA synthase; CODH, carbon monoxide dehydrogenase; BCL, benzoate-CoA ligase; BCR, benzoyl-CoA reductase; Hdr-IFO, heterodisulfide reductase-associated ion-translocating ferredoxin oxidoreductase; Dch, cyclohexa-1,5-diene-1-carboxyl-CoA hydratase; Had, 6-hydroxycyclohex-1-ene-1-carbonyl-CoA dehydrogenase; Oah, 6-oxycylohex-1-ene-1-carbonyl-CoA hydrolase; HPDhd, 3-hydroxypimeloyl-CoA dehydrogenase; PimB, acyl-CoA acetyltransferase; GcdH, glutaryl-CoA dehydrogenase; EHt, enoyl-CoA hydratase; 3-HbcdH, 3-hydroxyacyl-CoA dehydrogenase; ETF, electron transport flavoprotein; TCA, tricarboxylic acid.

A complete anaerobic benzoate oxidation pathway was discovered in the genomes of DscP3 and Dsc4 (Fig. 4). This is the first record of a complete anaerobic benzoate degradation pathway found in a *Chloroflexi* genome. While it was previously reported that the subphylum II *Chloroflexi* member DEHJ10 from Aarhus Bay contains subunits of BCR (52), our finding is the first record of complete BCR genes in members of subphylum I.

The first step in anaerobic benzoate degradation is benzoate-CoA ligase, which catalyzes the conversion of benzoate to benzoyl-CoA. The second step is the reductive dearomatization of benzoyl-CoA to cyclohex-1,5-diene-1-carbonyl-CoA with reduced ferredoxin by benzoyl-CoA reductase (BCR) (64). After we discovered BCR in the single-cell genomes of DscP3 and Dsc4, we reanalyzed SAG An-B16 from the Okinawa Trough and found that this SAG also contains these genes (15). Amino acid sequence and gene order comparisons revealed the BCRs of these marine *Chloroflexi* to be of class I (*bzdNOPQ*), the same class as previously found in DEHJ10 and most similar to the BCRs of the well-studied facultative *Azoarcus* spp. and *Thauera aromatica* (Fig. S7) (14, 65, 66). In contrast to the ATP-independent reversible class II BCRs (*bamBCDEFGHI*) found in *Syntrophus aciditrophicus*, *Desulfococcus multivorans*, *Geobacter* spp., and other obligate anaerobic benzoate degraders, class I BCRs are unlikely participants in benzoate fermentation, because the amount of energy available from benzoate oxidation is insufficient to support the energy requirement of the ATP-dependent class I BCR (64, 67–69). Rather, class II BCRs are found associated with anaerobic benzoate oxidation coupled with iron, sulfate, or proton reduction. The sole known exception is the hyperthermophilic archaeon *Ferroglobus placidus*, which utilizes a class I BCR despite its growth via iron reduction (70). It has been hypothesized that for the class II BCRs, Hdr-IFO allows for the generation of reduced ferredoxin to provide some of the necessary reducing power for benzoyl-CoA reduction (45).

10.1128/mBio.02022-17.6 FIG S5  Comparison of Rnf genes. Download FIG S5, DOCX file, 0.3 MB. Copyright © 2017 Sewell et al. 2017 Sewell et al. This content is distributed under the terms of the Creative Commons Attribution 4.0 International license.

10.1128/mBio.02022-17.7 FIG S6  Phylogenetic analysis of Hdr-IFO. Download FIG S6, DOCX file, 0.1 MB. Copyright © 2017 Sewell et al. 2017 Sewell et al. This content is distributed under the terms of the Creative Commons Attribution 4.0 International license.

10.1128/mBio.02022-17.8 FIG S7  Comparison of benzoyl-CoA reductase genes. Download FIG S7, DOCX file, 0.1 MB. Copyright © 2017 Sewell et al. 2017 Sewell et al. This content is distributed under the terms of the Creative Commons Attribution 4.0 International license.

In the anaerobic benzoate degradation pathway, cyclohex-1,5-diene-1-carbonyl-CoA is subsequently metabolized either to 2,3-didehydro-pimeloyl-CoA (using the *Rhodospeudomonas palustris* BadK-BadH-BadI pathway) or to 3-pimeloyl-CoA (using the Dch-Had-Oah pathway) (64). The genomes of DscP3, Dsc4, and An-B22 contain all of the genes required for the degradation of benzoyl-CoA to 3-hydroxypimeloyl-CoA, including those for a benzoate-CoA ligase (*bzdA*), the four subunits of *Azoarcus-*type BCR (*bzdNOPQ*), ferredoxin (*bzdM*), and the BCR ferredoxin-regenerating system (*korAB* and *bzdV*), as well as genes for the modified β-oxidation pathway (*bzdWXY* and *dch-had-oah*).

Further oxidation of 3-hydroxypimeloyl-CoA produces three molecules of acetyl-CoA and CO_2_. Many microorganisms contain multiple acyl-CoA reductase and enoyl-CoA hydratase family proteins that might participate in the degradation of 3-hydroxypimeloyl-CoA (71). In *Azoarcus* sp. strain EbN1, multiple gene clusters have been identified encoding enzymes for β-oxidation (72). Therefore, any of the acyl-CoA reductase and enoyl-CoA hydratase genes within the genomes of DscP3 and Dsc4 could be responsible for the oxidation of 3-hydroxypimeloyl-CoA.

The genome of DscP3 contains genes for electron transfer flavoproteins (Etfβα) and an Etf-linked acyl-CoA dehydrogenase (ACAD) with an Etf dehydrogenase (*fixABCX*) system. ACADs catalyze the reversible oxidation of acyl-CoA to a 2,3-enoyl-CoA involved in β-oxidation (73). While members of an acyl-CoA dehydrogenase family share the same reaction mechanism and a high degree of sequence identity, they differ widely in their substrate-binding specificity. The nonbifurcating acyl-CoA dehydrogenase-Etf system has mostly been studied in mammals in which electrons are transferred from fatty acid-oxidizing ACADs to a membrane Etf-quinone oxidoreductase (Etf-QO) that transfers the electrons to the quinone pool (74). In members of *Clostridium*, Etfαβ is known to form a stable complex with a propionyl-CoA dehydrogenase (Pcd) or butyryl-CoA dehydrogenase (BcdA) (75, 76). The BcdA-Etfβα complexes of *Firmicutes* are bifurcating, coupling the NADH-dependent exergonic reduction of crotonyl-CoA to butyryl-CoA to the endergonic reduction of ferredoxin (Fd). The latter is key for energy conservation by the formation of either NADH via Rnf or hydrogen via Ech (76, 77). An exception is the Pcd-Etfβα complex of *Clostridium propionicum*, in which a 15-amino-acid insertion into the Etf α subunit is proposed to keep the protein in a conformation which catalyzes the NADH-dependent reduction of acryloyl-CoA to propionyl-CoA but does not support the reduction of Fd (75). The *fixABCX* system, which has been described in several syntrophic and diazotrophic bacteria, contains an Etfβα homologue, FixAB, and is proposed to bifurcate electrons from NADH to Fd, FixX, and the Etf-quinone oxidoreductase homologue, FixC, with subsequent electron transfer to the quinone pool (78). While the substrate of ACAD in DscP3 is unknown, Etf has been observed to be the electron acceptor for glutaryl-CoA dehydrogenase (79). Thus, we hypothesize that the electrons from the oxidation of glutaryl-CoA to crotonyl-CoA in benzoate degradation are confurcated with a reduced ferredoxin to form NADH or funneled to the quinone pool via the FixABCX system.

### (4) New insights into DscP2-2.

**FIG 5:**
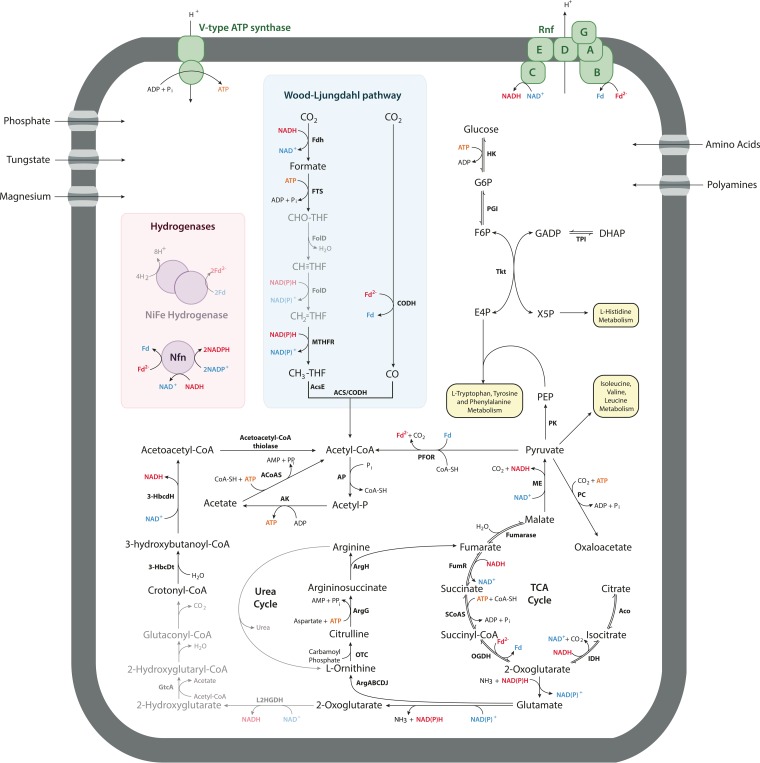
Metabolic pathways found in DscP2/DscP2-2. Genes identified in either DscP2 or DscP2-2 are shown in black, and those not found but hypothesized to be present are in gray. Proton-translocating complexes are in green, and electron-bifurcating complexes are in orange. Figure abbreviations are the same as in Fig. 4 with the addition of the following: ACoAS, acetyl-CoA synthase; IDH, isocitrate dehydrogenase; Aco, aconitase; Tkt, transketolase; E4P, erythrose-4-phosphate; G6P, glucose-6-phosphate; X5P, xylose-5-phosphate; HK, hexokinase; PGI, phosphoglucose isomerase; PC, pyruvate carboxylase; ArgA, *N*-acetylglutamate synthase; ArgB, acetylglutamate kinase; ArgC, *N*-acetylglutamylphosphate reductase; ArgD, *N*-acetylornithine aminotransferase; ArgE, acetylornithine deacetylase; OTC, ornithine transcarbamoylase; ArgG, argininosuccinate synthase; ArgH, argininosuccinate lyase; L2HGDH, l-2-hydroxyglutarate dehydrogenase; GtcA, glutaconate CoA-transferase.

Our previous analysis of the partial genome of DscP2, originating from two single cells from site 1230 (13), identified hydrogenase accessory proteins encoded by *hypABCDEF*, an archaeal-type H^+^ ATPase (subunits ABCDEFI), a [NiFe]-hydrogenase, and a partial F_420_-reducing hydrogenase and genes encoding the following enzymes required for the WLP: formate dehydrogenase, formyltetrahydrofolate synthetase, CO-dehydrogenase/CO-methylating acetyl-CoA synthase complex (α and β subunits), methyltetrahydrofolate reductase, and methylene tetrahydrofolate methyltransferase. Besides a 16S rRNA gene identical to DscP2, the here-reported DscP2-2 genome also shares 275 coding sequences (CDS) (>97% nucleotide identity) with DscP2, including carbon monoxide oxidoreductase/acetyl-CoA synthase from the WLP and the archaeal-type H^+^ ATPase (13). Moreover, the here-reported DscP2-2 genome contains 321 unique coding sequences, including an Rnf complex and genes homologous to the bifurcating NADH-dependent reduced ferredoxin:NADP^+^ oxidoreductase (Nfn) (Fig. 5).

As previously mentioned, partial genomes of DscP3 and Dsc4 contain genes encoding an Rnf complex. The genes for this Rnf complex in the subphylum I *Chloroflexi* are identical to the one found in DscP2-2. The presence of these genes in all three genomes indicates that they might have been shared via horizontal gene transfer (80). While this is not the first time that an Rnf complex has been found in a member of the phylum, it is the first reported observation of an Rnf complex in a member of *Chloroflexi* subphylum II (81).

The NADH-dependent reduced ferredoxin:NADP^+^ oxidoreductase (NfnBA) is a heterodimeric enzyme that catalyzes the reversible oxidation of NADH and ferredoxin with the reduction of NADP^+^ via flavin-based electron bifurcation (82). The [2Fe-2S]-containing NfnB subunit shows sequence similarities to bacterial dihydroorotate dehydrogenase (*pyrK*), and NfnA, which harbors two [4Fe-4S] clusters and flavin adenine dinucleotide (FAD), shows similarity to the small subunit of NADPH-dependent glutamate synthase (GltD) (83). Because of the similarities of the subunits to other functional genes and the inconsistency of nomenclature in genome annotations, their location on the genome is paramount to identifying their putative function. Homologues of *gltD* are found in all domains of life and are part of known or putative electron transfer systems (84). For example, NfnBA is often annotated as SudBA in the genomes of sulfate-reducing bacteria and archaea (85, 86), which catalyze the reduction of polysulfide to H_2_S using NADPH as electron donor. In DscP2-2, the genomic context vicinal to *nfnBA* looks remarkably similar to a conserved region near the origin of replication of *Dehalococcoides* spp. (Fig. S8) (87). In fact, an examination of *Dehalococcoides* and *Dehalogenimonas* genomes reveals putative *nfnBA* genes conserved in all members of the clade. The putative NfnBAs with highest amino acid similarity to members of *Chloroflexi* subphylum II are the acetate-oxidizing, sulfate-reducing *Deltaproteobacteria* member *Thermodesulforhabdus norvegica* and the acetate-oxidizing syntrophic firmicute *Syntrophaceticus schinkii*.

10.1128/mBio.02022-17.9 FIG S8  Phylogenetic analysis of NfnAB. Download FIG S8, DOCX file, 0.5 MB. Copyright © 2017 Sewell et al. 2017 Sewell et al. This content is distributed under the terms of the Creative Commons Attribution 4.0 International license.

## DISCUSSION

### Ecological niches and putative catabolisms of deep-sea *Chloroflexi*. (i) DscP3 and Dsc4.

We present here the first comprehensive metabolic insights into the abundantly present, uncultivated subphylum I *Chloroflexi* from deep-sea sediments based on single-cell genomic analyses. The genomes of DscP3 and Dsc4 were predicted to be 2 to 2.7 Mb in size and between 50 and 80% complete, respectively. While incomplete, the data obtained provide nevertheless a useful framework to predict central metabolic features of these populations and to speculate about their role in the deep-sea sediment environment. In DscP3 and Dsc4, we identified a complete Wood-Ljungdahl pathway with some metabolic features reminiscent of the pathway of homoacetogenesis of *Moorella thermoacetica*, including an electron-bifurcating HdrABC-MvhD-MetVF methylene tetrahydrofolate reductase and an NADP^+^-dependent methenyltetrahydrofolate cyclohydrolase/methylene tetrahydrofolate dehydrogenase. In contrast, however, evidence for energy conservation via Rnf and/or Hdr-IFO rather than by Ech, as in *M. thermoacetica*, was found. We found no supporting evidence for H_2_ as electron donor for the reductive Wood-Ljungdahl pathway; however, the presence of hydrogenases cannot be ruled out, based on the incompleteness of the genome. Instead, the presence of three formate dehydrogenases as well as of a formate transporter suggests that formate may play a central role and serve as electron donor in homoacetogenic metabolism of DscP3 and Dsc4. Based on the available data, we predict that DscP3 and Dsc4 are capable of both a heterotrophic and an autotrophic, homoacetogenic metabolism (Fig. 4). Heterotrophic homoacetogenesis would rely on glycolysis or possibly benzoate oxidation to provide the reducing equivalents for the WLP. For autotrophic homoacetogenesis with formate, three of the four formates could be used as electron donors where electron equivalents from formate oxidation are bifurcated via NAD-FdhAB and/or HdrBCA-FdhBA to 1.5 molecules of NADH and 1.5 molecules of reduced ferredoxin; one reduced ferredoxin is used for CO_2_ reduction to CO, and 0.5 molecule of reduced ferredoxin is oxidized via Rnf to 0.5 molecule of NADH (net 2 molecules of NADH). The two NADH molecules are used in the HdrABC-MvhD-MetVF reaction for reduction of methylene tetrahydrofolate and a low-redox-potential carrier, which could be NADP or Fd. If NADPH is formed, it is consumed in the methylene tetrahydrofolate dehydrogenase reaction. If Fd is the acceptor, formation of NADH for methylene tetrahydrofolate dehydrogenase by Rnf could result in further energy conservation, although this scenario is less likely as we did not find evidence for the presence of Nfn. Since these genomes aIso contain genes encoding a complete glycolytic pathway, a *M. thermoacetica*-type metabolism of fermenting glucose to three acetate molecules is conceivable if carbohydrates are the catabolic substrates.

The finding of a complete *Azoarcus*-type pathway for anaerobic benzoate degradation to acetyl-CoA in conjunction with the WLP is interesting but not unprecedented. The iron-reducing euryarchaeon *Ferriglobus placidus* contains both a class I benzoyl-CoA reductase and a WLP but lacks acetate kinase (see Table S2 in the supplemental material). In DscP3 and Dsc4, this combination of pathways would enable fermentation of 1 benzoate molecule to 3.75 acetate molecules according to the equation C_7_H_5_O_2_^−^ + 4H_2_O + 0.5HCO_3_^−^ → 3.75CH_3_COO^−^ + 2.25H^+^, where the reductive reactions of the Wood-Ljungdahl pathway accept the reducing equivalents from benzoate oxidation to acetate. However, under standard-state conditions, this reaction is endergonic by +12 kJ/mol benzoate and thus proceeds only if the environmental acetate concentration is low, as found in sites 1227, 1229, and 1230, e.g., as mediated by acetate-consuming sulfate-reducing bacteria (18). The finding of an ATP-consuming class I but not of a reversible class II benzoyl-CoA reductase complicates the energetics of this proposed benzoate fermentative pathway; however, the Fd requirement could be met by reverse electron transport via the Hdr-IFO complex, which is directly downstream of the genes encoding the benzoyl-CoA reductase. The presence of both Rnf and Hdr-IFO in the genomes of these deep-sea *Chloroflexi* is not without precedent, as *Desulfovibrio vulgaris* also encodes both ion-translocating complexes, which are expressed under different growth conditions (46–48). The findings of a presumably catabolic Wood-Ljungdahl pathway, including the energy-conserving enzymes, as well as of an anaerobic benzoate degradation pathway are consistent with the geochemically identified richness in organic matter of the sites studied (14, 15, 18).

10.1128/mBio.02022-17.11 TABLE S2  Metabolic comparisons. Download TABLE S2, DOCX file, 0.1 MB. Copyright © 2017 Sewell et al. 2017 Sewell et al. This content is distributed under the terms of the Creative Commons Attribution 4.0 International license.

The finding in DscP3 and Dsc4 of a presumably functional formate:quinol oxidoreductase closely related to the FDH-like protein CISM from *Dehalococcoides/Dehalogenimonas* spp. is of evolutionary significance. The unique catabolism of the latter microorganisms consists of reductive dehalogenation of an organohalogen with H_2_ as electron donor. *Dehalococcoides/Dehalogenimonas* spp. are strictly anaerobic, obligate reductively dehalogenating *Chloroflexi* with a small, 1.5-Mbp genome (11, 88). The evolutionary origin of these highly niche-specialized terrestrial microorganisms, including the metabolic core; the mode of energy conservation associated with reductive dehalogenation, the origin of reductive dehalogenases, and the mechanism of electron transfer between the HupL hydrogenase and the membrane-associated reductive dehalogenases are unknown and the focus of much speculation. Proteomic data and recent biochemical data indicated that the FDH-like protein CISM is part of a larger protein complex coupling these two enzymatic reactions (11, 12, 53). The FDH-like protein is predicted to be nonfunctional as a formate dehydrogenase, based on the absence of a selenocysteine or cysteine in a key catalytic position and the inability of *Dehalococcoides/Dehalogenimonas* spp. to utilize formate as electron donor. In DcP3 and Dsc4, FdnGHI may be involved in detoxification reactions of reactive oxygen species as observed in *E. coli* and *Bacillus* sp. (56, 58, 99). Our finding of the closest match of a *Dehalococcoides/Dehalogenimonas*-type CISM protein to an FdnGHI formate dehydrogenase in another *Chloroflexi* member links *Dehalococcoides/Dehalogenimonas* spp. to homoacetogenic deep-sea *Chloroflexi* and provides an important missing link for understanding the origin and the metabolic core of terrestrial *Dehalococcoides/Dehalogenimonas* as well as reductive dehalogenation.

### (ii) DscP2-2.

In previous work, we found in the genome of the DscP2 population of deep-sea sediment *Chloroflexi* an incomplete Wood-Ljungdahl pathway, consisting of a formate dehydrogenase, formyltetrahydrofolate synthetase, CO-dehydrogenase/CO-methylating acetyl-CoA synthase complex (α and β subunits), methyltetrahydrofolate reductase, and methylene tetrahydrofolate methyltransferase, as well as evidence for an [NiFe]-hydrogenase and a partial F_420_-reducing hydrogenase (13). Besides a 16S rRNA gene identical in sequence to DscP2, the here-reported DscP2-2 genome also shares 275 coding sequences (>97% nucleotide identity) with DscP2, including carbon monoxide oxidoreductase/acetyl-CoA synthase from the WLP and the archaeal-type H^+^ ATPase (13). Consistent with the 1.38-Mb genome size previously reported for DscP2, we estimate the genome of DscP2-2 to be 1 to 1.3 Mb in size (13). The DscP2-2 genome reported here contains 321 unique coding sequences, including genes encoding an Rnf complex and genes homologous to the bifurcating Nfn (Fig. S8). These new findings substantially expand our understanding of the catabolism of the DscP2 population and the deep-sea *Chloroflexi* as a group, as we identified here the genes for putative energy conservation. We predict for the DscP2 population an H_2_-dependent homoacetogenesis and energy conservation via Rnf. H_2_ may be oxidized with NADP^+^, and NADPH may be bifurcated by Nfn to form reduced ferredoxin and NADH. Reduced ferredoxin may be oxidized to NADH by Rnf and coupled to electrogenic H^+^ or Na^+^ translocation. NADH is then reoxidized by the reductive reactions of the Wood-Ljungdahl pathway.

In conclusion, single-cell genomic data from this study provide important new, in particular bioenergetic, insights, and substantiate homoacetogenesis as the main catabolism in deep-sea sediment *Chloroflexi*. The phylum *Chloroflexi* was so far not known to be capable of a homoacetogenic lifestyle. Furthermore, the finding of a predicted functional FDH that is the closest match to the nonfunctional FDH-like protein CISM of *Dehalococcoides/Dehalogenimonas* spp. provides the first insights into the origin of the niche-specialized, terrestrial reductively dehalogenating *Chloroflexi*.

There is currently no evidence that the genomic composition observed for DscP3, Dsc4, and DscP2 evolved in deep-sea sediments as a result of adaptive evolution; it is more likely that DscP3, Dsc4, and DscP2-2 are present at low abundance in surface sediments or in anoxic niches in the seawater column above these sediments and function there in anaerobic organic matter oxidation. Their particular homoacetogenic metabolism, perhaps in conjunction with a general characteristic of growth and resilience to decay, may explain the relative abundance maintenance of these *Chloroflexi* in the respective deep-sea sediments.

## MATERIALS AND METHODS

### Cell sorting, genome amplification, and sequencing.

Deep-sea sediment samples from Peruvian Margin sites 1227, 1229, and 1230, provided by Jennifer Biddle, at sediment depths 0.3, 1, and 7.3 mbsf, respectively, were collected during the IODP Leg 201 and stored, frozen, at −80°C without glycerol preservation for 8 years. Cell sorting, cell lysis, and multiple displacement amplification (MDA) of the single-cell genomes were performed at the Bigelow Laboratory Single Cell Genomics Center (https://scgc.bigelow.org/) as described previously (13). 16S rRNA sequence analysis with the Ribosomal Database Project (RDP) (89) revealed 4 wells containing *Chloroflexi* 16S rRNA sequences. The MDA products yielded 25, 26, 35, and 36 ng/µl of DNA for wells 657A03, 657K04, 662N06, and 653N14, respectively, after cleanup with the Zymo ZR DNA sequencing cleanup kit. Samples were sent to GeneWiz (South Plainfield, NJ) for sequencing with a 2- by 150-bp paired-end (PE) Illumina HiSeq 2500 sequencer (San Diego, CA) using the Nextera XT DNA library preparation.

### Assembly and contaminant screening.

Quality measurements of raw reads were performed using FastQC (https://www.bioinformatics.babraham.ac.uk/projects/fastqc/). Reads were quality filtered with Trimmomatic 0.36 with a quality threshold of 20 and a minimum sequence length of 52 bases. Quality-filtered reads were assembled using SPAdes 3.10.0 with the –sc option and kmer sizes 21, 25, 33, 37, 43, 47, 51, 55, 59, 63, 67, 71, 73, 79, 81, and 85. Contaminant screening was performed using the Automated Contamination Detection and Confidence (ACDC) software (90), which categorizes contigs based on GC content and tetranucleotide frequency. The assembly of well 657K04 was found to have two 16S rRNA gene sequences, indicating that two cells may have been sorted and amplified in one well. Each of the 16S rRNA sequences was identical to single-cell genomes 657A03 and 662N06, respectively. Using the ACDC software (90), we observed that tetranucleotide frequencies and GC content were sufficient to separate the two genomes in assembly 657K04 (see Fig. S2 in the supplemental material). Reads from 657K04 were mapped back to the contigs using Bowtie 2 (91), extracted using SAMtools (92), and coassembled with the reads from either well 657A03 or well 662N06. The combined assemblies of 657A03 and 657K04 are here referred to as DscP3 (deep-sea single cell, population 3). Since the 16S rRNA sequence of 662N06 is identical to DscP2 (13), the combined assembly of 662N06 and 657K04 is referred to as DscP2-2. According to the same naming convention, genomic content from well 653N14 is Dsc4.

### Genome annotation and estimation of completeness.

Putative coding sequences (CDS) were determined and annotated using Prokka (93) and compared to the annotations obtained using Rapid Annotations using Subsystem Technology (RAST) (94) and BLAST. Some computationally assigned annotations were manually changed based on orthologs in related genomes, neighboring genes, and protein domain searches with InterPro (https://www.ebi.ac.uk/interpro/) and Pfam (95).

### Accession number(s).

All assemblies have been made available to the public in the Integrated Microbial Genomes (IMG) database under the indicated accession numbers: DscP2-2, 154118; DscP3, 154115; Dsc4, 154419.

10.1128/mBio.02022-17.1 TEXT S1  Supplemental references. Download TEXT S1, DOCX file, 0.03 MB. Copyright © 2017 Sewell et al. 2017 Sewell et al. This content is distributed under the terms of the Creative Commons Attribution 4.0 International license.
