# Correction for Sewell et al., “Homoacetogenesis in Deep-Sea *Chloroflexi*, as Inferred by Single-Cell Genomics, Provides a Link to Reductive Dehalogenation in Terrestrial *Dehalococcoidetes*”

**DOI:** 10.1128/mBio.00312-18

**Published:** 2018-03-06

**Authors:** Holly L. Sewell, Anne-Kristin Kaster, Alfred M. Spormann

**Affiliations:** a Department of Civil and Environmental Engineering, Stanford University, Stanford, California, USA; b Institute for Biological Interfaces (IBG 5), Karlsruhe Institute of Technology, Karlsruhe, Germany; c Department of Chemical Engineering, Stanford University, Stanford, California, USA

## AUTHOR CORRECTION

Volume 8, issue 6, e02022-17, 2017, https://doi.org/10.1128/mBio.02022-17. The text erroneously referred to the catalytic subunit of FdnGHI as FdnI rather than the correct FdnG, and it refers to the integral membrane, quinone-binding subunit as FdnG instead of FdnI. This reversal of the accepted naming convention of the subunits is consistent throughout the text, figures, and supplemental information. Thus, every instance of FdnI should be read as referring to FdnG and vice versa. This clarification does not affect a major finding of this study, namely, that the catalytic subunit of a putative formate dehydrogenase-N, FdnG, from DscP3 and Dsc4 has the highest sequence identity to the complex iron-sulfur molybdoenzyme (CISM) from *Dehalococcoides* spp.

Furthermore, “*Dehalococcoidetes*” should be replaced by “*Dehalococcoidia*.”

