# Supplementary material for: Homoacetogenesis in Deep-Sea Chloroflexi, as Inferred by Single-Cell Genomics, Provides a Link to Reductive Dehalogenation in Terrestrial Dehalococcoidetes
Source: mBio. 2017 Dec 19;8(6):e02022-17. doi: 10.1128/mBio.02022-17 (PMC5736913; doi:10.1128/mBio.02022-17)
Supplement: FIG S2 [file mbo006173645sf2.docx]

##
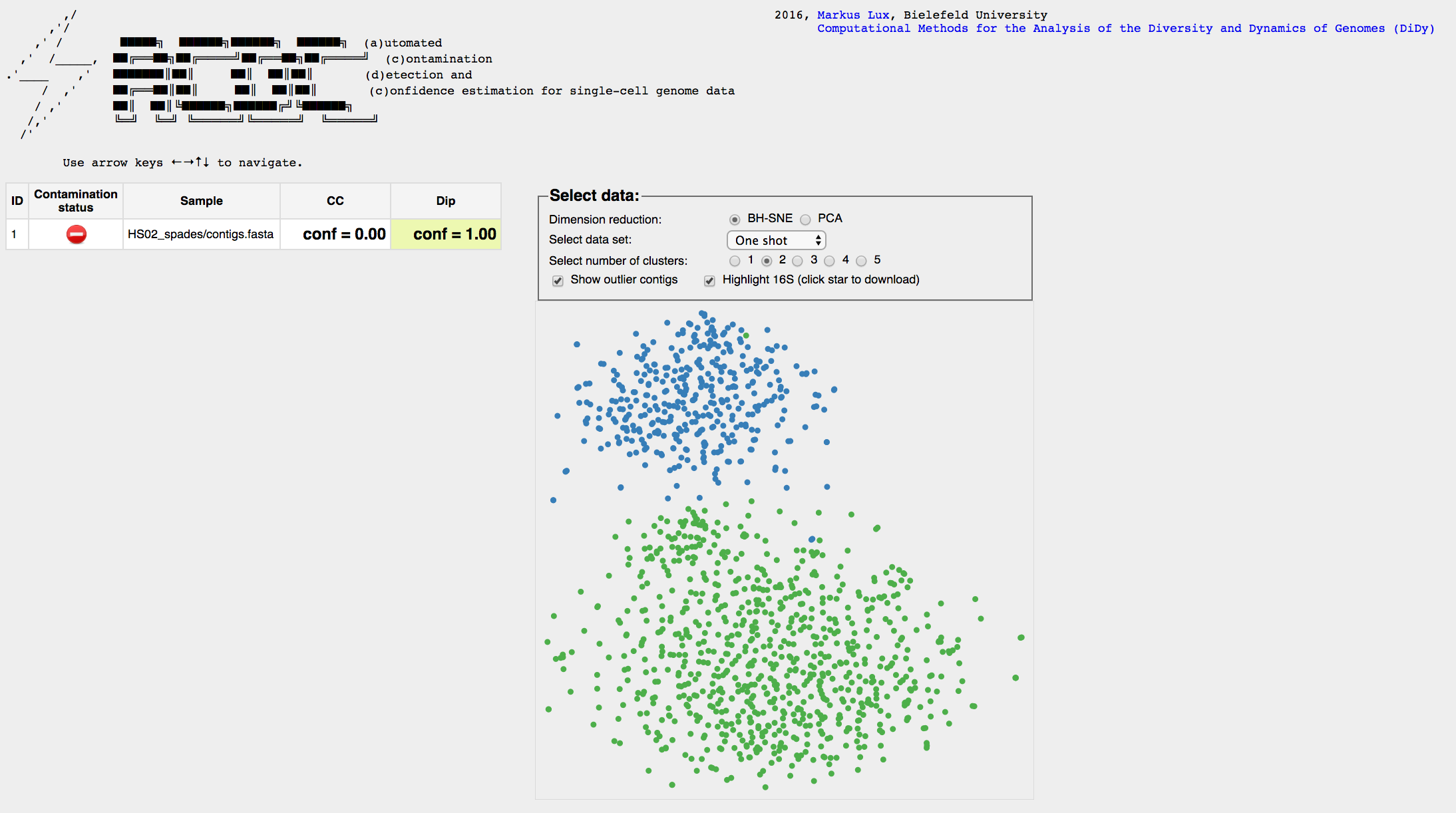


## Figure S2: Contamination Screen

The genomic content from well 657K04 had two different 16S sequences, one identical to single cell 657A03 and the other to 662N06, indicating that there were two cells amplified in the well. ACDC [[1](#Lux16)] was used to identify the contigs that belonged to each genome using tetranucleotide frequencies and GC content. The figure to the left shows a Barnes-Hut Stochastic Neighbor Embedding (BH-SNE) plot of this data. The two colors represent the two genomes, blue being the organism most closely related to 662N06 and green to 657A03. Raw reads were mapped back on to these contigs, extracted and reassembled with either the reads from 657A03 or 662N06 providing a moderate improvement to the assembly statistics.
