# Supplementary material for: Homoacetogenesis in Deep-Sea Chloroflexi, as Inferred by Single-Cell Genomics, Provides a Link to Reductive Dehalogenation in Terrestrial Dehalococcoidetes
Source: mBio. 2017 Dec 19;8(6):e02022-17. doi: 10.1128/mBio.02022-17 (PMC5736913; doi:10.1128/mBio.02022-17)
Supplement: TABLE S1 [file mbo006173645st1.docx]

Table S1: Assembly statistics
