# Supplementary material for: Homoacetogenesis in Deep-Sea Chloroflexi, as Inferred by Single-Cell Genomics, Provides a Link to Reductive Dehalogenation in Terrestrial Dehalococcoidetes
Source: mBio. 2017 Dec 19;8(6):e02022-17. doi: 10.1128/mBio.02022-17 (PMC5736913; doi:10.1128/mBio.02022-17)
Supplement: FIG S3 [file mbo006173645sf3.docx]

##

## Figure S3: Sequence Alignment of Formate Dehydrogenase-Like Enzymes

Protein sequences of formate dehydrogenase-like proteins in Dsc4, *Dehalogenimonas lykanthroporepellens*, and *Dehalococcoides ethenogenes* 195 aligned using ClustalW and visualized with Geneious 10.0.7 (http://www.geneious.com) [[2](#Lar07),[3](#Kea12)]. Regions outlined in purple are protein domains annotated by InterPro [[4](#Hun09)]. PS51318; PROSITE TAT signal domain, PF04879; Molybdopterin oxidoreductase Fe4S4 domain (Molybdop_Fe4S4), PF00384, Molybdopterin oxidoreductase domain, PF01568; Molybdopterin dinucleotide binding domain (Molydop_binding).
