# Supplementary material for: Homoacetogenesis in Deep-Sea Chloroflexi, as Inferred by Single-Cell Genomics, Provides a Link to Reductive Dehalogenation in Terrestrial Dehalococcoidetes
Source: mBio. 2017 Dec 19;8(6):e02022-17. doi: 10.1128/mBio.02022-17 (PMC5736913; doi:10.1128/mBio.02022-17)
Supplement: FIG S4 [file mbo006173645sf4.docx]

##
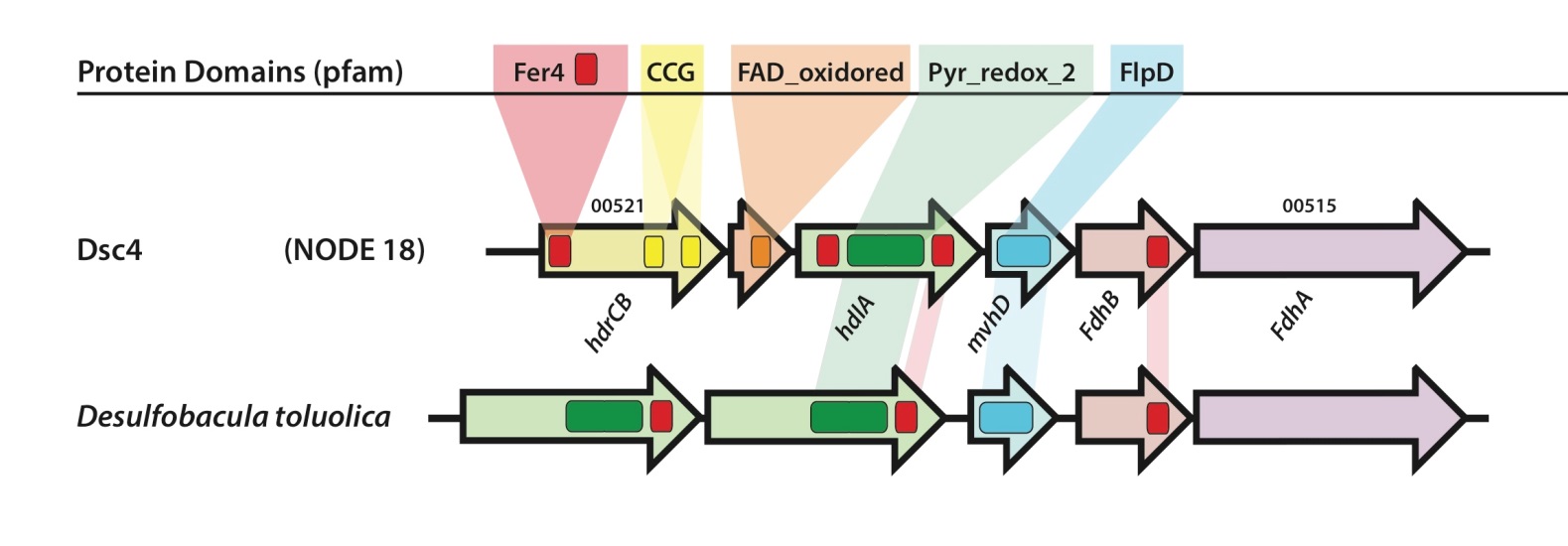


## Figure S4: Heterodisulfide Reductase-Associated Formate Dehydrogenase

Comparison of heterodisulfide reductase-associated formate dehydrogenases from Dsc4 and *Desulfobacula toluolica*. Protein families are annotated as follows: Fer4 (red) is the 4Fe-4S binding domain, PF00037; FAD_oxidored (orange) is the pterin binding protein family, PF12831; CCG (yellow) is the cysteine-rich domain, PF02754; Pyr_redox_2 (green) is the pyridine nucleotide-disulfide oxidoreductase family which contains a small NAD(P)H binding domain within a larger FAD domain, PF07992; FlpD (blue) is the methyl-viologen reducing hydrogenase delta subunit family containing 4 conserved cysteine residues [[5](#Fin161)].
