# Supplementary material for: Homoacetogenesis in Deep-Sea Chloroflexi, as Inferred by Single-Cell Genomics, Provides a Link to Reductive Dehalogenation in Terrestrial Dehalococcoidetes
Source: mBio. 2017 Dec 19;8(6):e02022-17. doi: 10.1128/mBio.02022-17 (PMC5736913; doi:10.1128/mBio.02022-17)
Supplement: FIG S5 [file mbo006173645sf5.docx]

## Figure S5: Comparison of Rnf Genes

A) Maximum Likelihood tree of concatenated protein alignments of RnfACDEG constructed using PhyML 3.0 [[6](#Gui101)]. Branches are colored by the order of genes in the operon as indicated in B) [[7](#Bie11)].
