# Supplementary material for: Homoacetogenesis in Deep-Sea Chloroflexi, as Inferred by Single-Cell Genomics, Provides a Link to Reductive Dehalogenation in Terrestrial Dehalococcoidetes
Source: mBio. 2017 Dec 19;8(6):e02022-17. doi: 10.1128/mBio.02022-17 (PMC5736913; doi:10.1128/mBio.02022-17)
Supplement: FIG S6 [file mbo006173645sf6.docx]

## Figure S6: Phylogenetic analysis of Hdr-IFO

PhyML-constructed maximum Likelihood tree of concatenated nucleotide alignments of the heterodisulfide reductase ion-translocating ferredoxin oxidoreductase (Hdr-IFO) [[6](#Gui101)].
