# Supplementary material for: Homoacetogenesis in Deep-Sea Chloroflexi, as Inferred by Single-Cell Genomics, Provides a Link to Reductive Dehalogenation in Terrestrial Dehalococcoidetes
Source: mBio. 2017 Dec 19;8(6):e02022-17. doi: 10.1128/mBio.02022-17 (PMC5736913; doi:10.1128/mBio.02022-17)
Supplement: FIG S7 [file mbo006173645sf7.docx]

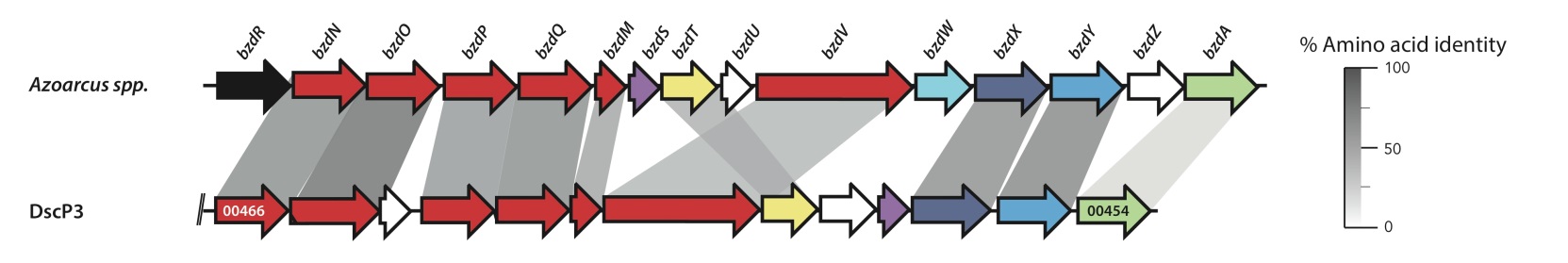


## Figure S7: Comparison of Benzoyl-CoA Reductase Genes

Comparison of benzoyl-CoA reductase and benzoate oxidation pathway genes in DscP3 and *Azoarcus* spp. Genes with the same color are predicted to have the same function. Grey shaded lines between the two gene order representations show BLASTp amino acid identity comparisons.
