# Supplementary material for: Homoacetogenesis in Deep-Sea Chloroflexi, as Inferred by Single-Cell Genomics, Provides a Link to Reductive Dehalogenation in Terrestrial Dehalococcoidetes
Source: mBio. 2017 Dec 19;8(6):e02022-17. doi: 10.1128/mBio.02022-17 (PMC5736913; doi:10.1128/mBio.02022-17)
Supplement: FIG S8 [file mbo006173645sf8.docx]

## Figure S8: Phylogenetic analysis of NfnAB

A) Maximum Likelihood tree of concatenated alignments of NfnAB and homologues. Branches are colored by phyla. B) Comparison of neighboring gene order in DscP2-2 and *Dehalococcoides mccartyi* VS (DhcVS_22-36). Grey shaded lines between the two gene order representations show BLASTp amino acid identity comparisons as determined by BLASTp as implemented using EasyFig [[8](#Sul11)]. Unlabeled genes encode for hypothetical. gmk, guanylate kinase; pitA, phosphate/sulfate transporter; trk, cation transport operon; comEC and comEA, competence proteins.
