# Supplementary material for: Homoacetogenesis in Deep-Sea Chloroflexi, as Inferred by Single-Cell Genomics, Provides a Link to Reductive Dehalogenation in Terrestrial Dehalococcoidetes
Source: mBio. 2017 Dec 19;8(6):e02022-17. doi: 10.1128/mBio.02022-17 (PMC5736913; doi:10.1128/mBio.02022-17)
Supplement: TEXT S1 [file mbo006173645s1.docx]

# Works Cited

x

| 1 | Lux, M. ACDC - Automated Contamination Detection and Confidence estimation for single-cell genome data. *BMC Bioinformatics* (2016), 1-11. |
| --- | --- |
| 2 | Larkin, M A, Blackshields, G, Brown, N P et al. Clustal W and Clustal X version 2.0. *Bioinformatics*, 23, 21 (2007). |
| 3 | Kearse, M, Moir, R, Wilson, A et al. Geneious Basic: an integrated and extendable desktop software platform for the organization and analysis of sequence data. *Bioinformatics*, 28, 12 (2012), 1647-1649. |
| 4 | Hunter, Sarah, Apweiler, Rolf, Attwood, Teresa K et al. InterPro: the integrative protein signature database. *Nucleic Acids Research*, 37 (2009), D211-D215. |
| 5 | Finn, R D, Coggill, P, Eberhardt, R Y et al. The Pfam protein families database: towards a more sustainable future. *Nucleic Acids Research (2016)*, 44 (2016), D279-D285. |
| 6 | Guindon, S, Dufayard, J F, Lefort, V, Anisimova, M, Hordijk, W, and Gascuel, O. New Algorithms and Methods to Estimate Maximum-Liklihood Phylogenies: Assessing the Performance of PhyML 3.0. *Systematic Biology*, 59, 3 (2010), 307-321. |
| 7 | Biegel, E, Schmidt, S, González, J M, and Müller, V. Biochemistry, evolution and physiological function of the Rnf complex, a novel ion-motive electron transport complex in prokaryotes. *Cell. Mol. Life Scie*, 68, 613 (2011). |
| 8 | Sullivan, Mitchell J, Petty, Nicola K, and Beatson, S A. Easyfig: a genome comparison visualiser. *Bioinformatics*, 27, 7 (2011), 1009-1010. |

x
